# Preparation of Plastics- and Foaming Agent-Free and Porous Bamboo Charcoal based Composites Using Sodium Silicate as Adhesives

**DOI:** 10.3390/ma14102468

**Published:** 2021-05-11

**Authors:** Weisheng Chai, Liang Zhang, Wenzhu Li, Min Zhang, Jingda Huang, Wenbiao Zhang

**Affiliations:** 1 School of Engineering, Zhejiang A&F University, Hangzhou 311300, China; cws1995@stu.zafu.edu.cn (W.C.); chaselz520103@163.com (L.Z.); lwz@zafu.edu.cn (W.L.); zhang888@rish.kyoto-u.ac.jp (M.Z.); 2 Research Institute for Sustainable Humanosphere, Kyoto 606-8501, Japan

**Keywords:** bamboo charcoal, sodium silicate, composites, pores

## Abstract

Plastics and foaming agents are often used to prepare large-size and low-density bamboo charcoal (BC) based composites. In this study, a plastic-free and foaming agent-free BC based composite was prepared by substituting sodium silicate (SS) for plastics. The effect of both the BC particle sizes and the usage amount of SS on the mechanical and adsorptive properties of the BC/SS composites were investigated. The experimental results show that when the BC particle size is 270 μm and the mass ratio of BC to SS is equal to 10:5, the BC/SS composite has the optimal foaming effect and best comprehensive properties. In addition, the foaming pores of the composite are caused by water vapor, which has difficulty escaping the BC because of the blockage of SS during the hot pressing process. In the BC/SS composite (10:5), the static bending intensity and the compressive strength reach respectively 6.13 MPa and 5.5 MPa, and the average pore size and porosity are 557.85 nm and 52.03%, respectively. In addition, its formaldehyde adsorptionrate reaches 21.6%. In view of good mechanical properties, formaldehyde adsorption, and environmentally friendly performance, the BC/SS composite has a great potential as a core layer of interior building materials.

## 1. Introduction

Bamboo charcoal (BC) is a solid residue generated by the pyrolysis of bamboo materials under anaerobic or hypoxic high-temperature conditions [1,2] and possesses abundant pore structure and high specific surface area, showing excellent functions in water purification [3], electrical conductivity [4], air filtration [5], soil improvement [6], photocatalysis degradation [7], formaldehyde adsorption [8], and sound insulation [9]. In daily life, BC is usually made into small-size particles with various shapes for air or water purification, but its scope of use is limited due to low usage amount and dust pollution. To address the above-mentioned shortcomings, large-size BC based composites have been developed in recent years [10]. Specifically, the BC/plastics composite board is widely reported due to its high mechanical strength and ability to partially replace wood and reduce the large consumption of forest resources, showing a great potential in building materials [11,12,13]. For example, the polylactic acid (PLA)/BC composite board could be prepared by mixing BC and PLA and has good mechanical, thermal, and optical properties in the range of BC content from 2.5 wt.% to 10 wt.% [14]. Li et al. [15] added electrically conductive BC nanoparticles to polyethylene (PE) with high molecular weight to prepare BC/PE composites, the resulting composites showed a segregated network structure and high conductivity, and they were adequate for many electrical applications. In addition, a thermoplastic polyester (PET) or polypropylene (PP)/BC composite could be obtained by mixing PET or PP with BC particles injecting, and then molding. And comparing with pure PET or PP, there is an obvious improvement for the mechanical properties [16].

From the above, we can see that BC mainly acts as a filler and plays a role in performance enhancement of BC/plastics; in other words, plastics could be used as adhesives to prepare BC-based composites [10]. However, plastics are suffering from these disadvantages such as flammability, easy aging, high cost, etc. [17]. Meanwhile, the pore structure of BC itself in the composites is easily blocked by plastics, leading to a reduction in its adsorption ability. Furthermore, plastics are highly polluting and recognized as one of the materials which are most difficult to degrade naturally [18]. Therefore, to improve the above-mentioned problems, large-size BC-based composites which are also environmentally friendly need to be developed.

Organic or inorganic binders are also commonly used for preparation of biomass- based composites. The traditional organic binders are of low curing temperature, but most of them would begin to decompose when the temperature exceeds 300 °C and seriously affect the strength of the composites [19]. Inorganic binders have lots of advantages, such as high temperature resistance, excellent durability, and little harm to the environment [20,21]. For example, aluminophosphate, an inorganic binder, synthesized by the reaction between Al(OH)_3_ and H_3_PO_4_, could be taken as adhesives and mixed with wood fibers to prepare wood-based boards, and the wood-based boards show good porosity and high strength, due to the addition of the aluminophosphate [22]. The phosphate-based adhesive could be also used to improve the strength of concrete [23,24]. The above inorganic adhesives show a good effect on improvement of the mechanical properties of biomass-based composites. Sodium silicate (SS), as a non-toxic, easy to prepare, low-cost, and good bonding inorganic adhesive, is also commonly used in these fields, such as refractory coatings, thermal insulation materials, castings, etc. [13]. For example, SS is used to modify 2017A-aluminum alloy to get a thick oxide functional surface with a good conductivity, high surface hardness, and outstanding wear resistance. In addition, SS mixed with sodium hydroxide could be used to improve the shear bond strength and compressive strength of the geopolymer [25,26,27]. Furthermore, SS is also able to act as a self-healing agent for some engineering composites [28]. From the above, we can see that SS is an excellent adhesive for preparation of biomass-based composites.

In conclusion, plastics could be used as adhesives for the preparation of BC based composites with high strength, but there is poor environmental performance. SS, as an inorganic adhesive, is almost pollution-free and as far as we know, SS is rarely reported in the preparation of BC based composites. Thus, in this study, a plastic-free and porous BC based composite is put forward by using SS as adhesives. The foaming mechanism of SS and the physical and chemical properties of the BC/SS composites were investigated. The BC/SS composite provides a new idea as a core layer of interior building materials.

## 2. Materials and Methods

### 2.1. Materials

BC particles with different sizes (1700 μm, 270 μm, 149 μm, and 74 μm) were purchased from Jiangshan Lvyi bamboo charcoal Co., Ltd., Zhejiang, China. Liquid sodium silicate (Na_2_O·nSiO_2_, modulus 2.25, solid contents of 30% (SiO_2_) and 13.75% (Na_2_O)), as adhesives, was purchased from Jiashan county refractory materials Co., Ltd., Zhejiang, China. Polytetrafluoroethylene (PTFE) films, 200 mm × 200 mm × 0.05 mm, were purchased from Shanghai Guoqiang rubber plastic Co., Ltd., Shanghai, China.

### 2.2. Preparation of BC/SS Composites

First, SS was treated by an ultrasonic equipment for 30 min and then mixed with BC particles in an agitator (SP-MDC60-5 dc electric mixer, dongguan zhiyu hardware technology co., LTD., Guangdong, China) with a stirring speed of 50 rap/min for 20 min. The mass ratio of BC to SS was set at 10:3, 10:4, and 10:5, respectively. Subsequently, the above mixture was poured into a stainless-steel mold (length × width × height = 150 mm × 150 mm × 20 mm) treated in advance with the PTFE film and preheated in an oven at 60 °C for 0.5 h and was then kept at 80 °C for 1.5 h until the mixture was fully solidified. Finally, the BC/SS composite was obtained after demolding and naturally cooling.

### 2.3. Porosity and Average Pore Size Test

The porosity and average pore size of the samples were tested by an autopore V 9600 automatic mercury injection device. The porosity (K) is estimated by the formula K = (1 − ρ_b_/ρ_a_) × 100%, where ρ_b_ stands for volume density, ρ_a_ for apparent density.

### 2.4. Formaldehyde Adsorption Performance Test

Before testing, the BC/SS composites were dried at 100 °C for 24 h. Subsequently, the composites were kept in a testing desiccator for 24 h and tested every 3 h. The formaldehyde adsorption rate is calculated by the formula that the formaldehyde adsorption rate (AJ) = (mt − mo)/mo × 100%, where mo and mt stand for the mass before and after adsorption, respectively.

### 2.5. Mechanical Properties and Aging Resistance Test

According to the physical and chemical properties test method of the Chinese standard “GB/T17657-2013” [29], the samples are processed into two sizes of 150 mm × 3 mm × 3 mm and 15 mm × 15 mm × 15 mm (length × width × height) by a table saw and used for the static bending strength test (the span of 100 mm and the compression rate of 3 mm/min) and the compressive strength test (the compression rate of 3 mm/min), respectively. The samples of each size were divided into 2 groups, each group for 6 samples. One group was directly used to test the static bending strength, elastic modulus, compressive strength, and compressive modulus of elasticity by a universal mechanical testing machine (598X, Instron company, Norwood, MA, USA). The other group was put into a constant temperature and humidity box with the relative humidity of 55% to conduct aging test for a week.

### 2.6. Characterization

Surface morphologies of the BC/SS composites were observed by a scanning electron microscope (SS-550, Shimadzu company, Tokyo, Japan) with an acceleration voltage of 15.00 KV. The composites were ground into powder and passed through a sieve with 200-mesh and then analyzed to detect crystal structure by an X-ray diffractometer (XRD) with a copper target, diffraction angle of 5–60°, and current intensity of 18 A. Chemical construction of the samples was detected using a Fourier infrared spectrometer (FTIR, Perkin Elmer, Akron, OH, USA) in the scanning range of 400–4000 cm^−1^, by scanning 32 times.

## 3. Results and Discussion

### 3.1. Forming Mechanism of BC/SS Composites

**Figure 1:**
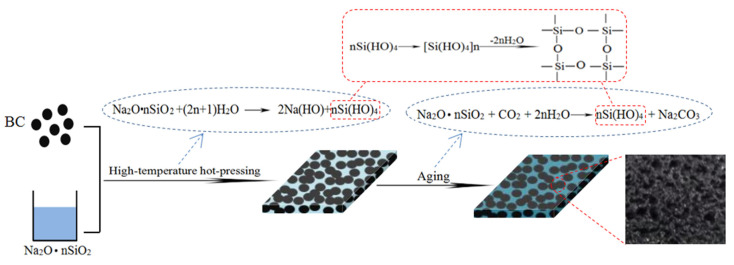
Preparation process and forming mechanism of BC/SS composites.

As shown in Figure 1, both BC and SS were uniformly mixed together and then pressed into BC/SS composites. SS acts as an adhesive and it was chosen because of its excellent advantages, such as waterproof, bacteria-proof, heat-resistant, acid-resistant, UV-resistant, and especially high-environmentally friendly performance [30]. In the hot-pressing and curing process, the main chemical reaction might be that Na_2_O·nSiO_2_ reacts with H_2_O and generates NaOH and Si(OH)_4_, which could be further dehydrated and condensed to produce a stabilized silicon three-dimensional structure affording strong adhesion [27]. During the aging process, the residual SS continued to react with CO_2_ and H_2_O in the air and to generate Na_2_CO_3_ and Si(OH)_4_, and the silicon three-dimensional structure was enhanced by the dehydration and condenses of Si(OH)_4_. When BC particles were in a certain size range, SS at the top of the composites solidified first and formed a relatively dense solidified layer. The water vapor in the inner of the composites did not have enough time to evaporate in the hot-pressing process, due to the internal resistance and bubble pores formed. In addition, the high viscosity of SS could make it difficult for the bubbles to escape [31]. In essence, SS, containing substantial quantities of moisture, also acts as a foaming agent under the condition of heating, so the porous BC/SS composites could be formed.

### 3.2. Effect of BC Particle Size on Formation of Composites

**Figure 2:**
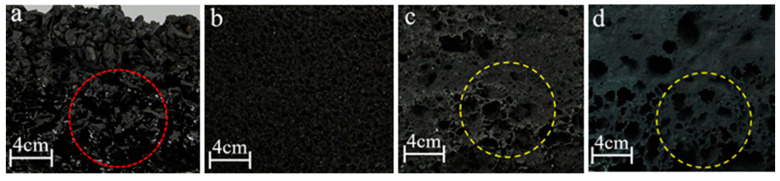
Effect of different BC particle sizes ((**a**) 1700 μm, (**b**) 270 μm, (**c**) 149 μm, (**d**) 74 μm) on the formation of the composites.

As shown in Figure 2, when the BC particle size was 1700 μm (mark “1700 μm-BC”), the distribution of SS in the composite was uneven at the mass ratio of 10:3~5 (BC/SS). The reason for this is that large-size BC particles would form large gaps among each other, which would allow SS to easily deposit to the bottom of the composite during the hot-pressing, leading to the lack of adhesives at the top of the composite and failure of formation (as shown in the red dotted line of Figure 2a) [32]. The composite with 270 μm-BC could be successfully prepared and shows uniform pore structure, which is because the smaller-size BC particles form smaller gaps and prevent SS from rapidly settling to its bottom during the hot-pressing, resulting in more uniform distribution (as shown in Figure 2b). At 149 μm- and 74 μm-BC, the composites show obvious bubbling and stratification (as shown in the yellow dotted line of Figure 2c,d), which might be a result of the gaps among BC particles being too small, making steam evaporation difficult during the hot-pressing. In a comprehensive consideration, the composite with 270 μm-BC is the optimal result and its properties and micro-structure would be studied in following.

### 3.3. Surface Morphologies

**Figure 3:**
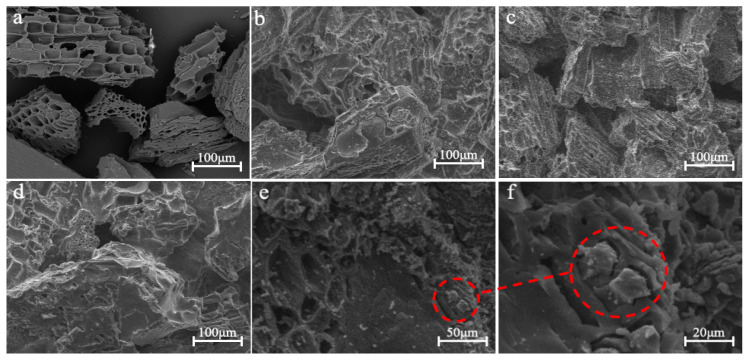
Micro-structures of the BC/SS composites with the mass ratios of (**a**) 10:0 (pure BC), (**b**) 10:3, (**c**) 10:4, (**d**) 10:5, and (**e**,**f**) its high-magnification images.

As shown in Figure 3a, BC particles are irregularly shaped and have an orderly arrangement of pore structure formed by bamboo conduits to the benefit of adsorption. As shown in Figure 3b–d, the composites with the mass ratios of 10:3~5 (BC/SS) show a similar surface structure, where gaps among BC particles are filled by SS which acts as an adhesive and forms a connection among BC particles to enhance the mechanical strength. However, the pore structure of BC would be mostly covered by SS, resulting in the possible decrease in adsorption properties. Figure 3e,f show high-magnification images of the micro structure of the composite (10:5). The BC particles are packaged by SS, but a part of the pore structure formed from bamboo conduits could still be seen (Figure 3e), this is one of the reasons for good adsorption properties. As shown in the red dotted line of the high-magnification images, some pores of BC particles are choked up by a nail formed from the solidified SS, meaning the water vapor generated in the composites during the hot-pressing could not escape.

### 3.4. Chemical Component Analysis

**Figure 4:**
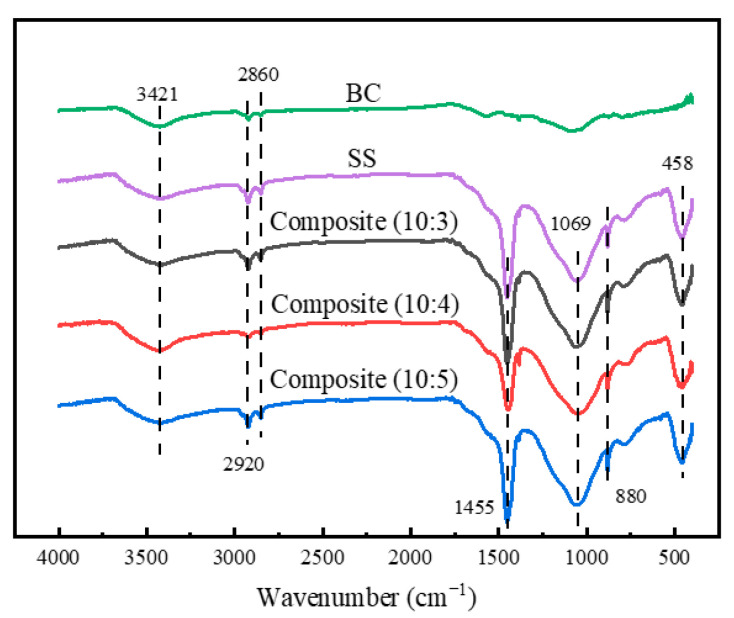
FTIR spectra of the BC/SS composites with different mass ratios.

The chemical structure of the composites was analyzed by FTIR, and the result is shown in Figure 4. Compared with pure BC, both the SS and BC/SS composites show a stronger absorption peak at 3421cm^−1^ due to the bending vibration of –OH bond [33], proving that a certain amount of moisture from SS stays in the interior of the composites and SS in the interior is incompletely dehydrated and solidified. The absorption peaks at 1100 cm^−1^, 800 cm^−1^, and 458 cm^−1^ are caused by the antisymmetric stretching vibration, symmetric stretching vibration, and bending vibration of the Si–O–Si bond, respectively [34]. The absorption peak at 1455cm^−1^ comes from CO_3_^2−^ and the asymmetric stretching of C–H bond shows two absorption peaks at 2860 cm^−1^ and 2920 cm^−1^, indicating that there is HCO^3−^ generating in the composites and corresponding to the speculation of the formation mechanism in Figure 1 [35,36], as well as illustrating that a part of SS is completely dehydrated and solidified in the hot-pressing and curing process. The solidification could improve the aging resistance and mechanical strength of the composites. Compared with pure BC and SS, the BC/SS composites have no new absorption peaks, proving that there is no chemical reaction between BC and SS, only physical bonding.

**Figure 5:**
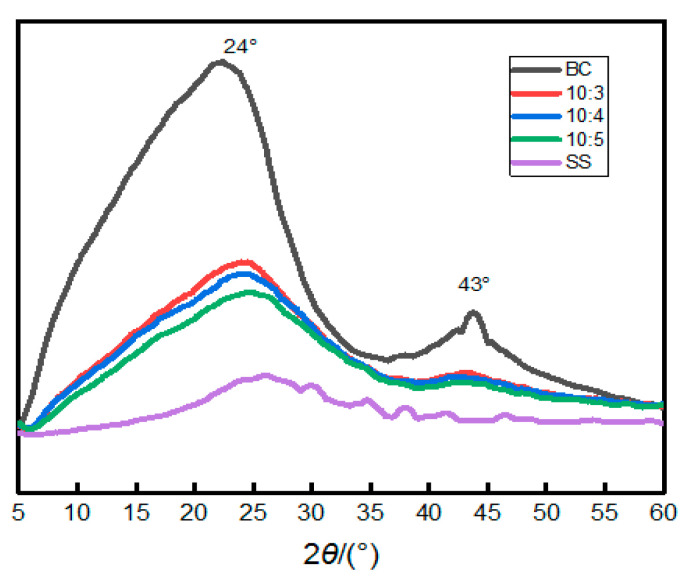
XRD patterns of the BC/SS composites with different mass ratios.

To test the change of their crystal degree, the composites were analyzed by XRD, with the pure BC as a contrast. As shown in Figure 5, the pure BC shows two diffraction peaks in the vicinity of 43° and 24°, respectively, which are caused by graphite crystallite crystal plane (002) and (001). The intensity of the diffraction peaks was reduced with the decrease in BC content, and the peak area gradually decreased. This is because the SS in the composites was not completely dehydrated and solidified, the solidification formed as a result of the water evaporation leads to the precipitation and full dehydration of the silica gel in the composite surface. The semi-solidified state of SS is conducive to improve the aging property and the mechanical strength of the composites.

### 3.5. Densities and Moisture Content

**Figure 6:**
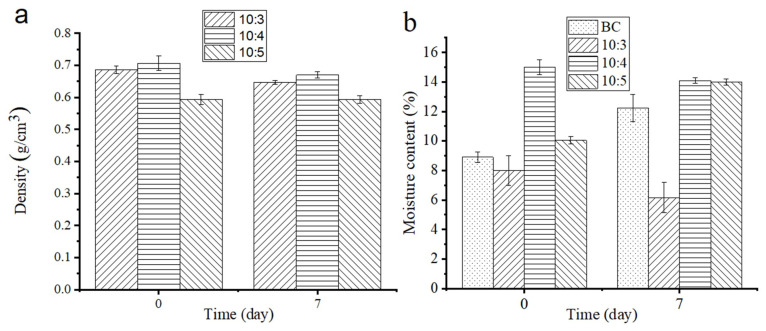
(**a**) Densities and (**b**) moisture content of the BC/SS composites with different mass ratios.

Normally, the densities of the BC/SS composites should increase with the increase in the SS proportion, due to a higher density of SS than BC, but it shows a different situation due to the influence of the foaming condition. As shown in Figure 6a, the densities of the composites first went up, and then down, with the increase in SS. This is because the foaming effect of the composites (10:3~4) was poor as a result of the low content of SS, and the density arose with the increase in SS. However, at the mass ratio of 10:5, the composite showed a good foaming effect, so the density decreased because a lot of water could escape through these foaming pores. At the mass ratio of 10:3~4, the densities of the composites were from 0.68 g/cm^3^ to 0.71 g/cm^3^, the minimum density appeared at (10:5) and was 0.59 g/cm^3^. After aging for seven days, the densities of the composites showed a similar change to that before aging, which depends on the content of SS and the foaming effect of the system. (10:5) exhibited no large change because the moisture evaporation reached equilibrium to the moisture absorption, and the densities of (10:3~4) decreased, which is caused by moisture evaporation in the composites during the cooling and aging process.

As shown in Figure 6b, the moisture content of the pure BC was 8.9% and went up to 12.23% after aging by absorbing moisture from the air. The moisture content of the composites (10:3~5) shows a similar change in trend to their densities. This is because with the increase in SS content, the moisture content of the composites increased, so the moisture content of (10:4) was higher than that of (10:3). However, since (10:5) had a good foaming effect, most of the moisture in SS could be out of the pore holes formed from foaming, so even though the content of SS increased, the moisture content still decreased. The moisture content of both (10:3~4) during the cooling and aging process was gradually reduced, illustrating that the poor foaming is not conducive to the absorption of moisture. Therefore, during the seven-day aging process, the composites were mainly dehydrated and showed less hygroscopic function, so both the moisture content and the densities decreased at the same time. However, the density of (10:5) had no obvious change, which might be the reason that the good foaming of the composites (10:5) is beneficial to moisture evaporation in the curing process, resulting in lower density [37]. The composites absorb moisture in the air and the residual SS reacts with CO_2_ and H_2_O in the air, which makes adsorption and desorption reach a balance, explaining why compared with the others, the composite (10:5) had a more apparent response to the change in humidity was able to better absorb moisture. 

### 3.6. Porosity and Average Pore Size

**Figure 7:**
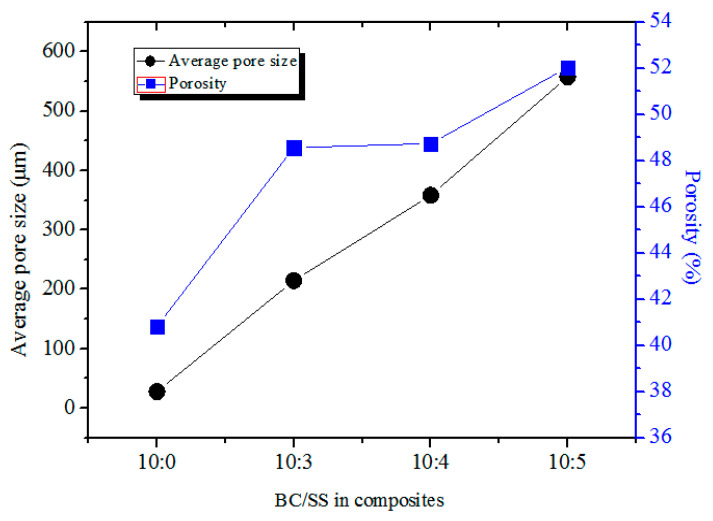
Average pore size and porosity of the composites with different BC/SS.

As shown in Figure 7, the porosity of the pure BC and the composites (10:3~5) went up with the increase in SS and was 40.82%, 48.56%, 48.74%, and 52.03%, respectively, indicating that with the increase in SS content, the foaming effect is gradually obvious. This is because SS acts as an adhesive and plays a role in a foaming agent, making the composites produce a certain number of pores. The average pore sizes of the composites show a similar changing trend to the porosity. With the addition of SS content, the average pore sizes of the composites became great, maximally reaching 557.85 nm at the one (10:5). This is because the moisture in the interior of the composites was more seriously blocked, preventing water vapor from smoothly venting and producing foaming pores during water evaporation. The average pore sizes of the composites became larger under the dual action of micropore plugging and foaming.

### 3.7. Formaldehyde Adsorption Performance

**Figure 8:**
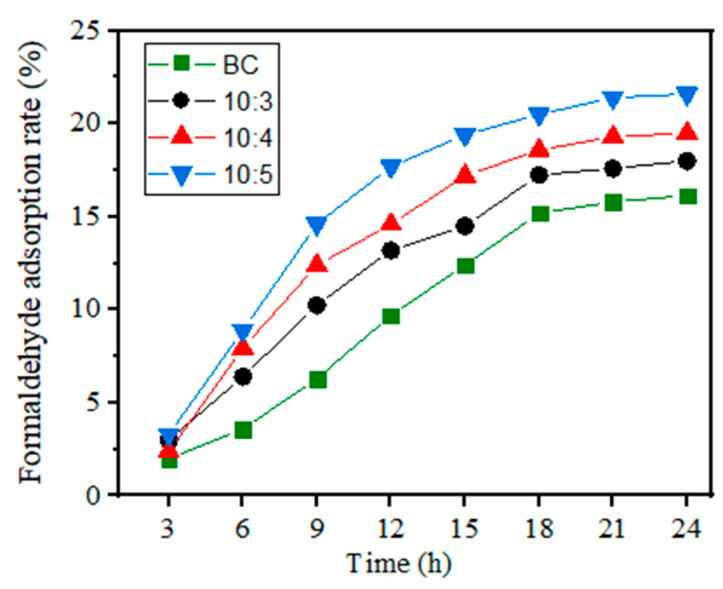
Formaldehyde adsorption rate curves of the composites with different BC/SS.

The formaldehyde adsorption capacity of the composites was estimated by a static adsorption test. As shown in Figure 8, the formaldehyde adsorption rate of the samples went up with time extension. The formaldehyde adsorption mainly occurred from 3 to 18 h, and gradually reached a saturation tendency after 18h, the formaldehyde adsorption rate of (10:5) was up to 21.6% at 24 h. Compared with the pure BC, the composites show a better adsorption ability for formaldehyde adsorption, and with the increase in SS, the static formaldehyde adsorption capacity gradually increased. This is because the increase in SS could improve the porosity of the composites and be good for formaldehyde adsorption.

### 3.8. Mechanical Performance

**Figure 9:**
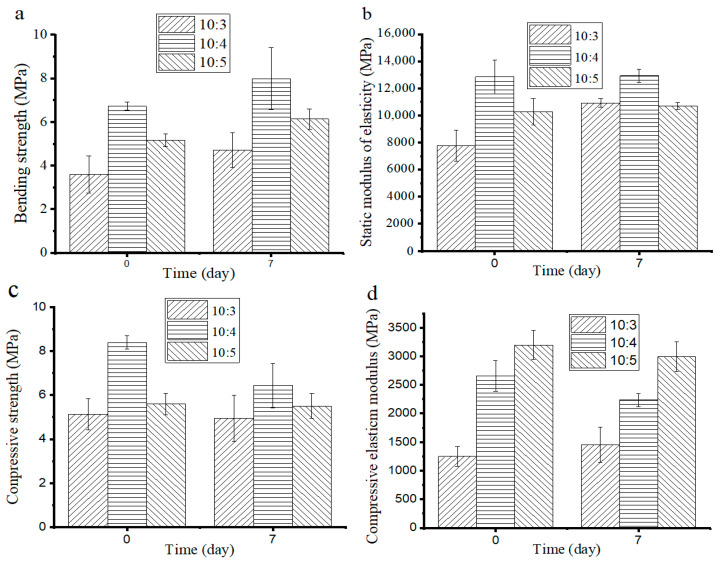
The (**a**) static bending strength, (**b**) static modulus of elasticity, (**c**) compressive strength, and (**d**) compressive elastic modulus of the composites.

As shown in Figure 9a, before aging, the static bending strength of the composites (10:3~5) was 3.59 MPa, 6.72 MPa, and 5.2 MPa, respectively. This is mainly related to the content of SS and the foaming status of the composites, corresponding to the change in trend of their densities. After aging for seven days, the static bending strength of all of the composites relatively enhanced and reached 4.5 MPa, 7.98 MPa, and 6.13 MPa, respectively. This is due to the high-intensitive hydrated silica gel, which is formed by the reaction between SS and CO_2_, as well as H_2_O in the air. The static bending strength of the composites had an improvement after aging, indicating that SS mainly causes dehydration and solidification during the hot-pressing and is conducive to the formation of the initial strength of the composites, followed by a sustainable reaction with CO_2_ in the air and further improving the strength of the composites. The static modulus of elasticity of the composites showed relatively little change (as shown in Figure 9b) before and after aging, indicating that the reaction between SS and CO_2_ could improve the strength of the composites and had little effect on the static modulus of elasticity.

Before aging, the compressive strength of the composites had a similar change as the static bending strength (as shown in Figure 9c) and was 5.15 MPa, 8.4 MPa, and 5.59 MPa, respectively. After aging, the static bending strength of all the composites decreased to 4.94 MPa, 6.44 MPa, and 5.5 MPa, respectively. (10:4) appears significantly lower at the static bending strength, which might be associated with the decrease in its density (as shown in Figure 6a). The compressive elastic modulus of the composites had no obvious change after aging (as shown in Figure 9d). However, the compressive elastic modulus was less than the static modulus of elasticity, indicating that the composites exhibit greater brittleness. While a certain plastic deformation was generated in the compression process, the deformation ability decreased with the increase in SS content. Overall, the 270 μm-BC and the mass ratio of 10:5 (BC/SS) are the optimal conditions for the preparation of the composites.

## 4. Conclusions

SS could be successfully used as an adhesive to prepare plastic-free BC/SS composites by mixing SS with BC and then hot-pressing. In addition, SS also plays the role of a foaming agent and makes the composites produce foaming pores due to the blasting of water vapor blockage by SS. According to the experimental results, both the BC particle size and the usage amount of SS have a significant effect on the forming effect and properties of the composites. At 270μm-BC and the mass ratio of 10:5 (BC/SS), the composite reached the optimal value and showed the most obvious foaming (a porosity of 52.03%) and the least density (0.59 g/cm^3^). With the increase in SS content, both the formaldehyde adsorption performance and the mechanical strength of the composite (10:5) increase and the maximum reached respectively 21.6% and 6.13 MPa after aging for seven days. This is due to the reinforcement of the solidification by the reaction between the residual SS and CO_2_, as well as H_2_O in the air. Due to simple preparation and good environmentally friendly performance, the composites could play a role in the adsorption and humidity control and be potential as a core layer of interior building materials.

## Data Availability

The data presented in this study are available on request from the corresponding author.

## References

[1] Mohan D., Sarswat A., Ok Y.S., Pittman C.U. (2014). Organic and inorganic contaminants removal from water with biochar, a renewable, low cost and sustainable adsorbent-a critical review. Bioresour. Technol..

[2] Li Y., Xing B., Ding Y., Han X., Wang S. (2020). A critical review of the production and advanced utilization of biochar via selective pyrolysis of lignocellulosic biomass. Bioresour. Technol..

[3] Li Z., Wang C., Lei T., Ma H., Su J., Ling S., Wang W. (2019). Arched bamboo charcoal as interfacial solar steam generation integrative device with enhanced water purification capacity. Adv. Sustain. Syst..

[4] Li S., Wang H., Chen C., Li X., Deng Q., Li D. (2018). Mechanical, electrical, and thermal properties of highly filled bamboo charcoal/ultra-high molecular weight polyethylene composites. Polym. Compos..

[5] Khandaker S., Toyohara Y., Kamida S., Kuba T. (2018). Adsorptive removal of cesium from aqueous solution using oxidized bamboo charcoal. Water Resour. Ind..

[6] Ye Y.J., Zhang Z.F. (2013). Research progress of the properties and application of bamboo charcoal. Appl. Mech. Mater..

[7] Wu F., Liu W., Qiu J., Li J., Zhou W., Fang Y., Zhang S., Li X. (2015). Enhanced photocatalytic degradation and adsorption of methylene blue via TiO^2^ nanocrystals supported on graphene-like bamboo charcoal. Appl. Surf. Sci..

[8] Chen Q., Zhu W., Li X., Lai S. (2009). Study on photocatalytic degradation of formaldehyde by TiO_2_ loaded on bamboo charcoal. J. Fujian Norm. Univ..

[9] Lin J.H., Lin C.M., Huang C.C., Lin C.C., Hsieh C.T., Liao Y.C. (2011). Evaluation of the manufacture of sound absorbent sandwich plank made of PET/TPU honeycomb grid/PU foam. J. Compos. Mater..

[10] Wang S., Zhang L., Semple K., Zhang M., Zhang W., Dai C. (2020). Development of biodegradable flame-retardant bamboo charcoal composites, Part I: Thermal and elemental analyses. Polymers.

[11] LI S., Wang H., Chen C. (2017). Size effect of charcoal particles on the properties of bamboo charcoal/ultra-high molecular weight polyethylene composites. J. Appl. Polym. Sci..

[12] Zhang H., Yao W., Qian S., Sheng K. (2018). Fabrication and reinforcement of ternary composites based on polypropylene matrix with bamboo particle/ultrafine bamboo-char. Polym. Compos..

[13] Chen Q., Zhang R., Qin D., Feng Z., Wang Y. (2018). Modification of the physical-mechanical properties of bamboo-plastic composites with bamboo charcoal after hydrothermal aging. BioResources.

[14] Ho M.P., Lau K.T., Wang H., Hui D. (2015). Improvement on the properties of polylactic acid (PLA) using bamboo charcoal particles. Compos. Part B Eng..

[15] Li S., Li X., Chen C., Wang H., Deng Q., Gong M., Li D. (2016). Development of electrically conductive nano bamboo charcoal/ultra- high molecular weight polyethylene composites with a segregated network. Compos. Sci. Technol..

[16] Lou C.W., Lin C.W., Lei C.H., Su K.H., Hsu C.H., Liu Z.H., Lin J.H. (2002). PET/PP blend with bamboo charcoal to produce functional composites. J. Mater. Process. Technol..

[17] Ragaert K., Delva L., Van Geem K. (2017). Mechanical and chemical recycling of solid plastic waste. Waste Manag..

[18] Burmeister M., Eilks I. (2014). A lesson plan to develop structured discussion of the benefits and disadvantages of selected plastics using the product-testing method. Sch. Sci. Rev..

[19] Zaretskiy L. (2016). Modified silicate binders new developments and applications. Int. J. Met..

[20] Burchenkova T., Slavkina V., Nelyub V. (2019). Modern technologies for the production of composites Based on inorganic binders. Mater. Today Proc..

[21] Rusati P.K., Song K.I. (2018). Magnesium chloride and sulfate attacks on gravel-sand-cement-inorganic binder mixture. Constr. Build. Mater..

[22] Chen T., Wu Z., Wang X.A., Wang W., Huang D., Wei Q., Wu B., Xie Y. (2018). Hierarchical lamellar aluminophosphate materials with porosity as ecofriendly inorganic adhesive for wood-based boards. ACS Sustain. Chem. Eng..

[23] Qiao J., Wang A., Li X. (2016). Preparation and performance of wheat-straw composite board with inorganic adhesive. BioResources.

[24] Ding Z., Lu Z.X., Li Y. (2011). Feasibility of basalt fiber reinforced inorganic adhesive for concrete strengthening. Advanced Materials Research.

[25] Polat A., Makaraci M., Usta M. (2010). Influence of SS concentration on structural and tribological properties of microarc oxidation coatings on 2017A aluminum alloy substrate. J. Alloys Compd..

[26] Morsy M.S., Alsayed S.H., Al-Salloum Y., Almusallam T. (2014). Effect of SS to sodium hydroxide ratios on strength and microstructure of fly ash geopolymer binder. Arab. J. Sci. Eng..

[27] Huang H., Ye G., Leung C., Wan K. Application of sodium silicate solution as self-healing agent in cementitious materials. Proceedings of the International RILEM Conference on Advances in Construction Materials through Science and Engineering.

[28] Phoo-ngernkham T., Maegawa A., Mishima N., Hatanaka S., Chindaprasirt P. (2015). Effects of sodium hydroxide and SS solutions on compressive and shear bond strengths of FA–GBFS geopolymer. Constr. Build. Mater..

[29] (2013). GB/T17657-2013, Test Methods of Evaluating the Properties of Wood-Based Panels and Surface Decorated Wood-Based Panels.

[30] Li X., Song W., Yang K., Krishnan N.A., Wang B., Smedskjaer M.M., Mauro J.C., Sant G., Balonis M., Bauchy M. (2017). Cooling rate effects in sodium silicate glasses: Bridging the gap between molecular dynamics simulations and experiments. J. Chem. Phys..

[31] Chen X., Lu A., Qu G. (2013). Preparation and characterization of foam ceramics from red mud and fly ash using sodium silicate as foaming agent. Ceram. Int..

[32] Koohestani B., Mokhtari P., Yilmaz E., Mahdipour F., Darban A.K. (2021). Geopolymerization mechanism of binder-free mine tailings by sodium silicate. Constr. Build. Mater..

[33] Rao A.P., Rao A.V., Pajonk G.M. (2007). Hydrophobic and physical properties of the ambient pressure dried silica aerogels with sodium silicate precursor using various surface modification agents. Appl. Surf. Sci..

[34] Innocenzi P. (2003). Infrared spectroscopy of sol-gel derived silica-based films: A spectra-microstructure overview. J. Non-Cryst. Solids.

[35] Li Y., Cheng X., Cao W., Gong L., Zhang R., Zhang H. (2015). Fabrication of adiabatic foam at low temperature with sodium silicate as raw material. Mater. Des..

[36] Chen Y., Hong Y., Zheng F., Li J., Wu Y., Li L. (2009). Preparation of silicate stalagmite from sodium silicate. J. Alloys Compd..

[37] Zhang J., Chen Q., You C. (2016). Biochar effect on water evaporation and hydraulic conductivity in sandy soil. Pedosphere.

